# Dynamic prediction of mortality after traumatic brain injury using a machine learning algorithm

**DOI:** 10.1038/s41746-022-00652-3

**Published:** 2022-07-18

**Authors:** Rahul Raj, Jenni M. Wennervirta, Jonathan Tjerkaski, Teemu M. Luoto, Jussi P. Posti, David W. Nelson, Riikka Takala, Stepani Bendel, Eric P. Thelin, Teemu Luostarinen, Miikka Korja

**Affiliations:** 1 Department of Neurosurgery, Helsinki University Hospital and University of Helsinki, Helsinki, Finland; 2 Analytics and AI Development Services, HUS IT Management, Helsinki University Hospital, Helsinki, Finland; 3 Department of Clinical Neuroscience, Karolinska Institutet, Stockholm, Sweden; 4 Department of Neurosurgery, Tampere University Hospital and Tampere University, Tampere, Finland; 5 Department of Neurosurgery, and Turku Brain Injury Centre, Turku University Hospital and University of Turku, Turku, Finland; 6 Section for Anesthesiology and Intensive Care, Department of Physiology and Pharmacology, Karolinska Institutet, Stockholm, Sweden; 7 Perioperative Services, Intensive Care Medicine and Pain Management, Turku University Hospital and University of Turku, Turku, Finland; 8 Division of Intensive Care, Department of Anesthesiology and Intensive Care, Kuopio University Hospital, Kuopio, Finland; 9 Department of Neurology, Karolinska University Hospital, Stockholm, Sweden; 10 Anaesthesiology and Intensive Care, Hyvinkää Hospital, Helsinki University Hospital and University of Helsinki, Helsinki, Finland

**Keywords:** Brain injuries, Outcomes research, Prognostic markers

## Abstract

Intensive care for patients with traumatic brain injury (TBI) aims to optimize intracranial pressure (ICP) and cerebral perfusion pressure (CPP). The transformation of ICP and CPP time-series data into a dynamic prediction model could aid clinicians to make more data-driven treatment decisions. We retrained and externally validated a machine learning model to dynamically predict the risk of mortality in patients with TBI. Retraining was done in 686 patients with 62,000 h of data and validation was done in two international cohorts including 638 patients with 60,000 h of data. The area under the receiver operating characteristic curve increased with time to 0.79 and 0.73 and the precision recall curve increased with time to 0.57 and 0.64 in the Swedish and American validation cohorts, respectively. The rate of false positives decreased to ≤2.5%. The algorithm provides dynamic mortality predictions during intensive care that improved with increasing data and may have a role as a clinical decision support tool.

## Introduction

Traumatic brain injury (TBI) is a leading cause of death and disability worldwide^1^. The global incidence is estimated to be 369–790 per 100,000 and the incidence is increasing^1,2^. Approximately 10% of patients with TBI require admission to an intensive care unit (ICU)^3^. Hospital and six-month mortality of patients with TBI treated in the ICU are approximately 15% and 20%, respectively^4^.

Increased intracranial pressure (ICP) is the main cause of TBI-related death. The degree of brain injury is indirectly characterized by the ICP and the derived cerebral perfusion pressure (CPP) (defined as mean arterial pressure [MAP] - ICP)^5–7^. Consequently, optimizing ICP and CPP is the cornerstone of modern treatment of severe TBI^8,9^. The recommended fixed ICP and CPP thresholds are <20–22 mmHg and 60–70 mmHg, respectively, although these thresholds may vary according to cerebrovascular reactivity, age and sex^10–12^. However, although the prognostic value of ICP and CPP is undisputed in terms of mortality, apart from static thresholds, the prognostic value of ICP and CPP has not been translated into a clinically useful tool that could be used to guide the treatment of individual TBI patients^5–7^. As there is a clear association between mortality and time spent above specific ICP thresholds, quantifying the effects of ICP and CPP on patient prognosis could aid clinicians to make more standardized and data-driven treatment decisions that are less affected by cognitive or personal biases, available resources and cultural factors^13^. Such measures would plausibly be particularly helpful when treatment periods are prolonged and clinical decision-making becomes increasingly challenging. Current prediction models in TBI are usually static, utilizing admission parameters and only result in an explained variance of about 35% in severe TBI^14,15^. Furthermore, TBI is a dynamic disease with lesion progression and subsequent deterioration in certain individuals, requiring longitudinally monitored data to optimize treatment.

One of the main challenges in translating machine learning applications to the bedside is the lack of external validation^16^. Here, we evaluate the external validity of the previously developed ICP-MAP-CPP algorithm in two international TBI cohorts after retraining the algorithm with additional available data in an extended training cohort^17^. To evaluate the performance of the algorithm, we calculated the area under the receiver operating characteristic curve (AUC) and precision recall curve (AUPRC), accuracy, false positives (i.e., the algorithm predicts death when the patient survives, which may lead to undertreatment) and false negatives (i.e., the algorithm predicts survival when the patient dies, which may lead to overtreatment). We calibrated the algorithm to minimize the number of false positives to avoid the worst-case clinical scenario of inaccurately withdrawing active life-saving treatment, as patients with severe TBI may recover remarkably well despite a poor initial prognosis^18^.

## Results

### Data and preprocessing

**Fig. 1:**
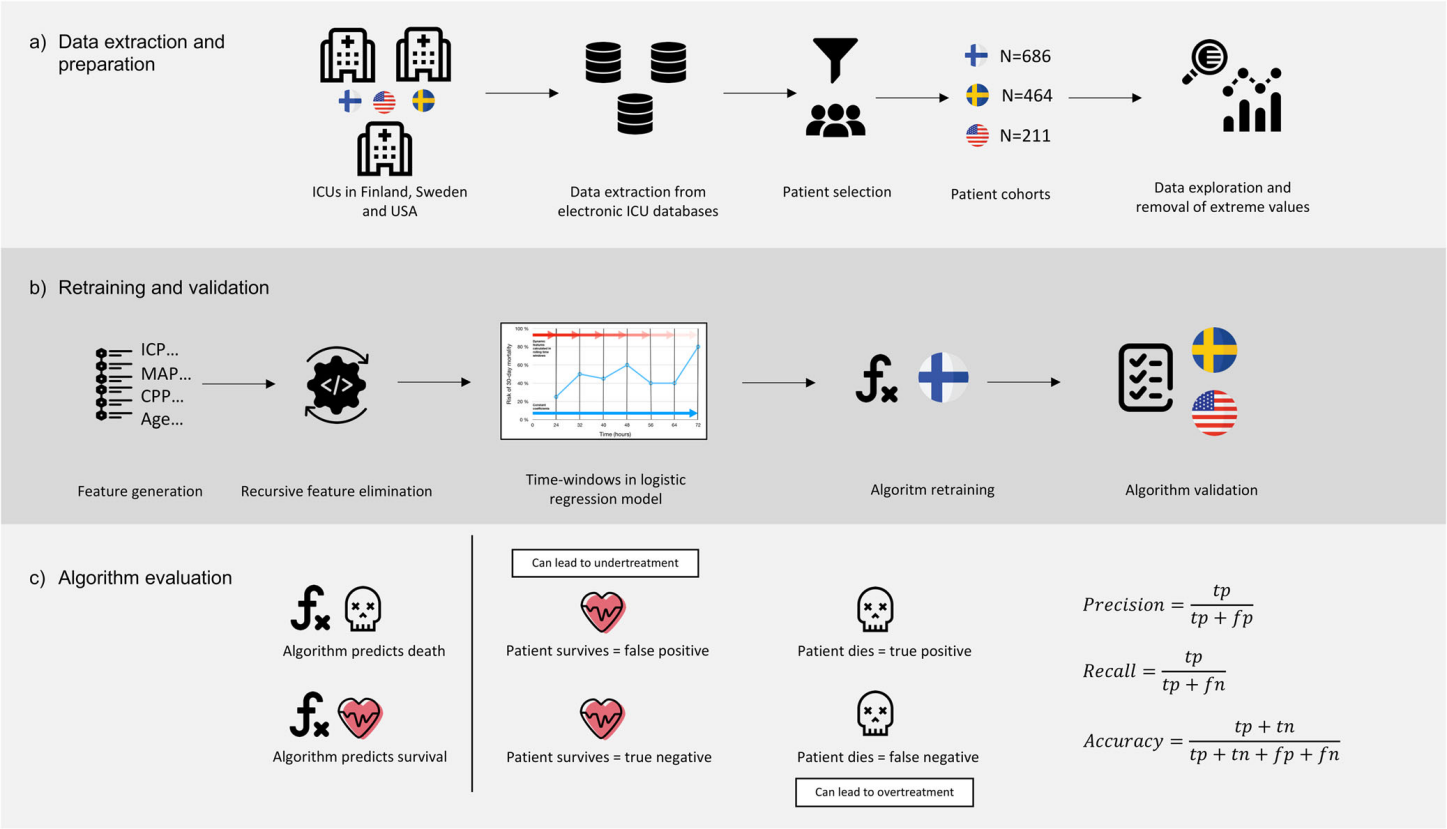
Summary of the workflow of the data extraction, preparation, retraining, validation and algorithm evaluation. Data were extracted from the electronic intensive care unit databases. Patients were filtered according to inclusion and exclusion criteria before forming the final patient cohorts. Data were explored and extreme measurements were removed (**a**).The model was based on intracranial pressure (ICP), cerebral perfusion pressure (CPP), mean arterial pressure (MAP) features and patient age. Following feature engineering and recursive feature elimination the final features were used in a time-window-based dynamic logistic regression model. The model was retrained in the Finnish patient cohort and validated separately in the Stockholm cohort and in the eICU cohort by assessing the area under the receiver operating characteristic curve, the area under the precision-recall curve, false positives, false negatives, and accuracy (**b**). Key algorithm evaluation metrics were the rate of false positives and false negatives, prevision, recall and accuracy (**c**).

We collected 1 to 5 min median values of ICP, MAP and CPP from electronic databases during the first 120 h following ICU admission (Fig. 1). As in the original study, we excluded extreme measurements (ICP > 100 mmHg, ICP < 0 mmHg, MAP > 150 mmHg, MAP < 20 mmHg) and did not impute missing values^17^. Missing values may occur frequently during intensive care and can themselves include important clinical information. If values were completely missing in a time window, the patient was excluded from that specific time window-based estimate.

### Patient characteristics

**Fig. 2:**
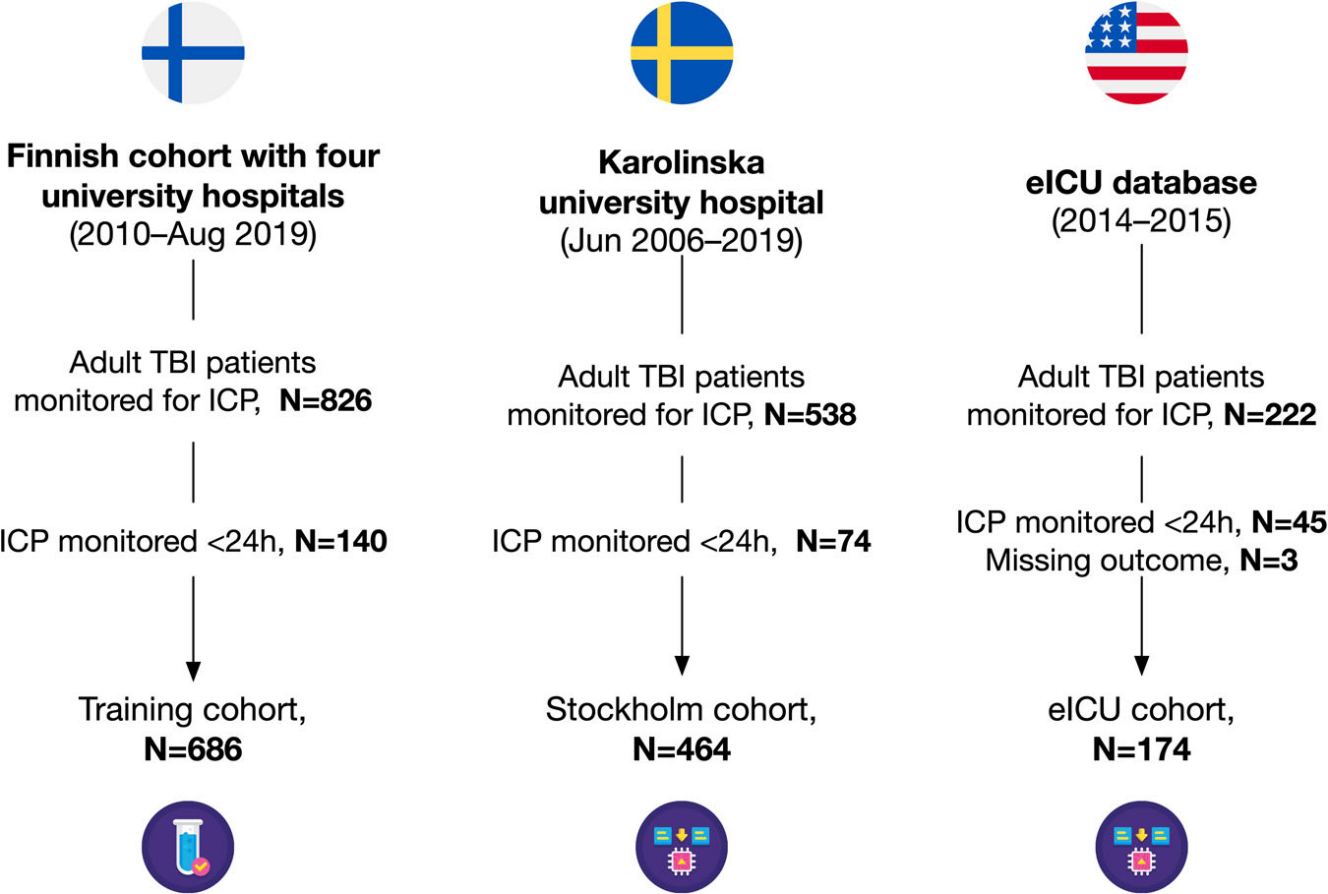
Study flow chart. The training cohort was collected from four university hospitals in Finland and the external validation cohorts were collected from Karolinska University Hospital (Stockholm, Sweden) and the eICU database.

The training cohort included 686 patients from four hospitals (Supplementary Table 1). 30-day mortality varied between 15% and 24% in the training cohort hospitals. The external validation cohorts consisted of 464 (Stockholm cohort) and 174 patients (eICU cohort), respectively (Fig. 2). The 30-day mortality rate was 17% in the training cohort and 13% in the Stockholm cohort. In-hospital mortality in the eICU cohort was 30%. Patient characteristics are shown in Tables 1 and 2. In short, patients in the Stockholm cohort were slightly older (median age 51 y vs. 46 y), were more frequently not obeying/localizing upon admission, had more frequently intracranial mass lesions >25 cm^3^, traumatic subarachnoid hemorrhages, epidural hematomas on their admission CT scans than patients in the Finnish training cohort (Table 1). Craniotomy for hematoma evacuation (61% vs. 40%) and the use of external ventricular drains (53% vs. 17%) were more frequent in the Stockholm cohort than in the training cohort, whereas decompressive craniectomies were less frequently performed in the Stockholm cohort (10% vs. 16%). Patients in the eICU cohort were younger (median age 38 vs 51 years), had lower GCS scores (prevalence of GCS 3–8 83% vs. 71%, had longer ICU length of stays (median 12 vs 10 days) and higher mortality rate (30% vs. 13%) than patients in the Stockholm cohorts (Table 2). The mean ICP level was higher in non-survivors than in survivors in all three cohorts during the first 120 h (Supplementary Fig. 1).

**Table 1:** Patient characteristics in the training cohort and in the Stockholm cohort.

Variables	Training cohort (*n* = 686)	Stockholm cohort (*n* = 464)	*p*-value
Age, median (IQR)	46 (28, 59)	51 (33, 62)	<0.001
Female sex	135 (20%)	106 (23%)	0.196
Admission GCS score^a^			
3 to 8	465 (68%)	321 (71%)	0.301
9 to 12	157 (23%)	87 (19%)	
13 to 15	64 (9%)	47 (10%)	
Admission motor score^b^			
None	186 (27%)	85 (20%)	<0.001
Extension	34 (5%)	52 (13%)	
Abnormal flexion	35 (5%)	52 (13%)	
Normal flexion	124 (18%)	72 (17%)	
Localizes/obeys	307 (45%)	156 (37%)	
Pupillary light reactivity^c^			
None react	32 (5%)	73 (16%)	<0.001
One reacts	100 (15%)	45 (10%)	
Both react	554 (80%)	325 (73%)	
Hypoxia^d^	110 (16%)	96 (23%)	0.004
Hypotension^e^	74 (11%)	16 (5%)	0.002
Marshall CT class^f^			
DI I	12 (2%)	0 (0%)	<0.001
DI II	260 (38%)	122 (26%)	
DI III	121 (17%)	77 (17%)	
DI IV	25 (4%)	14 (3%)	
EML/NEML	268 (39%)	249 (54%)	
Traumatic SAH^g^	491 (72%)	387 (84%)	<0.001
Epidural hematoma^h^	57 (8%)	66 (14%)	0.001
Craniotomy and hematoma evacuation	273 (40%)	281 (61%)	<0.001
Decompressive craniectomy	113 (16%)	48 (10%)	0.003
Primary	48 (7%)	39 (8%)	<0.001
Secondary	65 (9%)	9 (2%)	
External ventricular drain	114 (17%)	244 (53%)	<0.001
ICU length of stay (days), median (IQR)	9 (4, 14)	10 (5, 16)	0.021
30-day mortality	120 (17%)	60 (13%)	0.037

Hypoxia defined as any prehospital spO2 value <90%. Hypotension defined as any prehospital systolic blood pressure value mmHg.

*CT* Computer Tomography, *DI* Diffuse Injury, *EML* Evacuated Mass Lesion (>25 cm3), *NEML* Non-Evacuated Mass Lesion (>25 cm3), *GCS* Glasgow Coma Scale, *IQR* Interquartile Range.

^a^9 missing values in the Stockholm cohort.

^b^47 missing values in the Stockholm cohort.

^c^21 missing values in the Stockholm cohort.

^d^47 missing values in the Stockholm cohort.

^e^137 missing values in the Stockholm cohort.

^f^2 missing values in the Stockholm cohort.

^g^5 missing values in the Stockholm cohort.

^h^2 missing values in the Stockholm cohort.

**Table 2:** Patient characteristics in the eICU cohort.

Variable	eICU cohort (*n* = 174)
Age, median (IQR)	38 (24, 57)
Female sex	35 (20%)
Ethnicity	
Caucasian	132 (76%)
African American	20 (12%)
Hispanic	7 (4%)
Asian	2 (1%)
Native American	1 (1%)
Other/Unknown	11 (6%)
GCS score^a^	
3–8	129 (83%)
9–12	17 (11%)
13–15	10 (6%)
Motor score^a^, median (IQR)	
None	72 (46%)
Extension	7 (4%)
Abnormal flexion	9 (6)
Normal flexion	27 (17%)
Localizes/obeys	41 (27%)
APACHE IV score^b^, median (IQR)	75 (56, 90)
APACHE IV physiology score^b^, median (IQR)	82 (53, 87)
Length of ICU stay^b^, median (IQR)	12 (7, 19)
Length of hospital stay^b^, median (IQR)	18 (8, 27)
Hospital mortality	53 (30%)

*APACHE* acute physiology and chronic health evaluation, *ICU* intensive care unit, *IQR* interquartile range.

^a^GCS score defined as the worst within the first 24 h of admission according to the APACHE IV criteria. GCS score was missing for 19 patients.

^b^Missing for 16 patients.

### Training cohort and internal validation

Of the 55 evaluated features (Supplementary Table 2), 14 features were selected into the final retrained algorithm (Supplementary Fig. 2). Feature correlation is shown in Supplementary Fig. 3 and the features’ regression coefficients are shown in Supplementary Table 3. In the training cohort, the mean time of ICP monitoring was 90.7 h (SD 31.4) per patient.

**Fig. 3:**
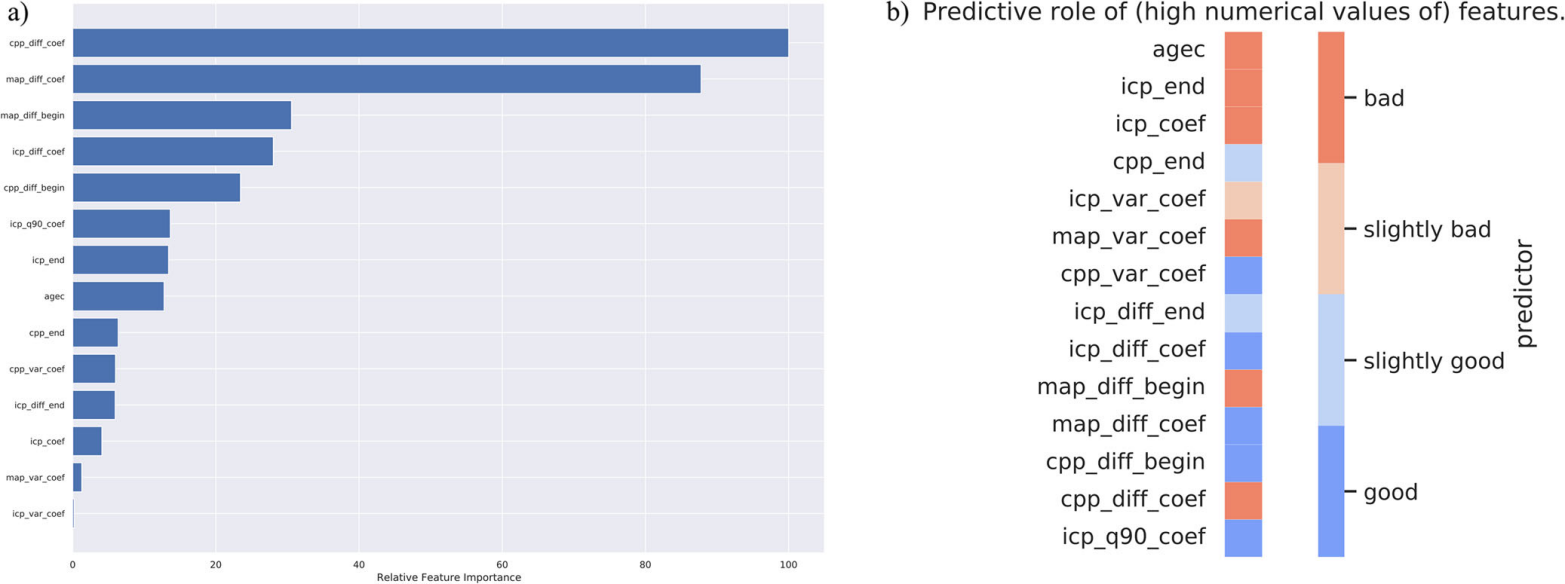
Relative feature importance and the direction of the predictions. The relative feature importance in descending order: cpp_diff_coef, map_diff_coef, map_diff_begin, icp_diff_coef, cpp_diff_begin, icp_q90_coef, icp_end, agec, cpp_end, cpp_var_coef, icp_diff_end, icp_coef, map_var_coef, icp_var_coef (**a**). Heat map showing the predictive role of included features (**b**). Red indicates that a higher feature value increases probability of death and blue indicates that a higher feature value increases probability survival. begin = mean value from the first derived 24-hour time-window; end = mean value from the last derived 8 h; coef = slope of the linear coefficient from the start of the derived time-window up to the time of the prediction; q90 = 90th percentile in the derived time-window; diff = mean of differences between consequent values in the derived time-window, var variance in the derived time-window, icp intracranial pressure, cpp cerebral perfusion pressure, map mean arterial pressure, agec age deciles.

The four most important features were the slope of the linear coefficient of the mean differences between two consequent CPP values in the derived time-window (cpp_diff_coef), the slope of the linear coefficient of the mean differences between two consequent MAP values in the derived time-window (map_diff_coef), the mean MAP from the first 24 h in relation to the mean differences between consequent MAP values in the derived time-window (map_diff_begin), the slope of the linear coefficient of the mean differences between two consequent ICP values in the derived time-window (icp_diff_coef) (Fig. 3).

**Fig. 4:**
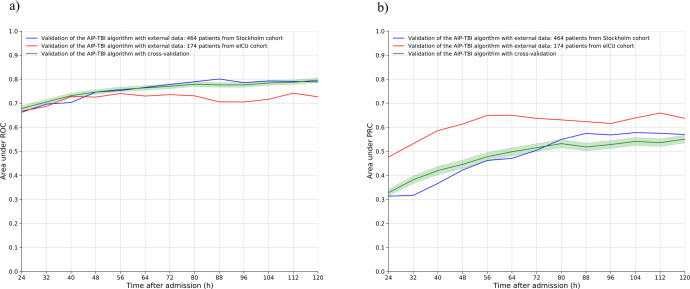
Time-dependent area under receiver operating curves (AUC) and area under the precision recall curves (AUPRC). Time-dependent AUCs (**a**) and time-dependent AUPRCs (**b**). The first AUC and AUPRC values are calculated after 24 h after which a new value is calculated for every 8 h. In green, the cross-validation results from the algorithm retraining, in blue, the results from the Stockholm cohort validation and in red, the results from the eICU cohort validation. The increase in AUC was more pronounced in the Stockholm cohort than in the eICU cohort, whereas the increase in AUPRC was more pronounced in the eICU cohort than in the Stockholm cohort.

In the training cohort (*n* = 686), the mean time of ICP monitoring was 90.7 h (SD 31.4) per patient. The AUC increased from 0.67 after the first 24 h to 0.79 after 120 h and the AUPRC increased from 0.33 after the first 24 h to 0.55 after 120 h (Fig. 4). The average fp and fn rates in all cross-validation repetitions decreased from 11.7% to 2.2% and remained stable between 11.7% and 13.1%, respectively (Supplementary Fig. 4). The accuracy (tp + tn/all) at 120 h was 85.4%.

### Stockholm cohort

**Fig. 5:**
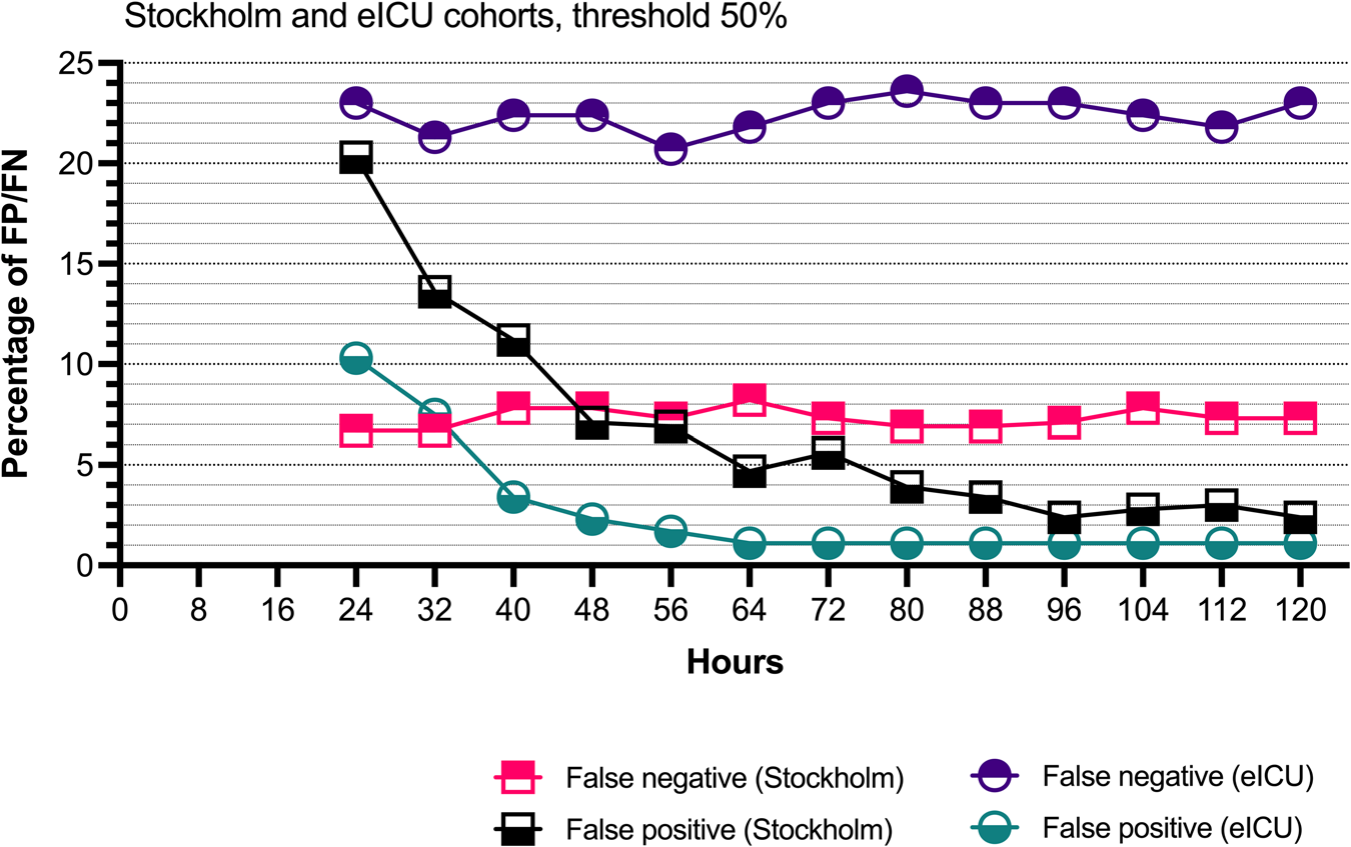
The false positive and false negative rates. The rates of false positives and false negatives as a function of time in the Stockholm cohort and in the eICU cohort, using a prediction threshold of 50%. The false positive rate drops notably with time indicating improved algorithm reliance as treatment gets prolonged and human clinical decision-making more challenging.

In the Stockholm cohort (*n* = 464), the mean time of ICP monitoring was 97.3 h (SD 31.9) per patient. The AUC increased from 0.66 after the first 24 h to 0.79 after 120 h. The AUPRC increased from 0.31 after the first 24 h to 0.57 after 120 h (Fig. 4). The fp rate decreased from 20.3% after 24 h to 2.4% at 120 h (Fig. 5). The fn rate remained stable at around approximately 7%. The accuracy at 120 h was 90.3%. Decreasing the 50% prediction threshold to 25% increased the fp rate but decreased the fn rate. In contrast, increasing the 50% prediction threshold to 75% decreased the rate of fp and increased the rate of fn (Supplementary Fig. 5). With a threshold of 50%, 55% of the false positives had an unfavorable functional outcome at 1-year after admission.

### eICU cohort

In the eICU cohort (*n* = 174), the mean time of ICP monitoring was 87.7 h (SD 34.3) per patient. The median hospital length of stay was 5 days (IQR 4–11 days) in patients who died and 21 days (IQR 16–30 days) in patients who survived. The AUC increased from 0.67 after the first 24 h to 0.73 after 120 h. The AUPRC increased from 0.48 after the first 24 h to 0.64 after 120 h (Fig. 4). The fp rate decreased from 10.3% to 1.1% and the fn rate remained stable at approximately 23% (Fig. 5). The accuracy at 120 h was 75.9%. Similarly, to the Stockholm cohort, decreasing the threshold increased the fp rate and increasing the threshold decreased the fp rate at the cost of a higher fn rate (Supplementary Fig. 6).

## Discussion

The ICP-MAP-CPP algorithm for dynamic mortality prediction in patients with TBI treated in the ICU displayed improving performance metrics in a time-dependent manner. In the Stockholm cohort, the AUC increased with time from 0.66 to 0.79 and the AUPRC from 0.31 to 0.57. In the eICU cohort, the corresponding metrics increased from 0.67 to 0.73 and from 0.48 to 0.64. The false positive rates were <2.5% in both cohorts and the false negative rates were 7% and 23%. In other words, fewer than 3 out of 100 patients survived when the algorithm predicted death and 7 to 23 out of 100 patients died despite the algorithm predicting survival. Thus, the accuracy of the algorithm was good in the Stockholm cohort (90%) and satisfactory in the eICU cohort (76%). Noteworthy, 55% of the false positives in the Stockholm cohort had unfavorable functional outcomes at 1 year. The potential use of the ICP-MAP-CPP algorithm is to alarm the clinician of changes in patient prognosis over the course of treatment, function as an objective decision-making support tool when trying to decide to continue or stop active treatment and inform relatives and next-of-kin. However, future prospective studies are needed to determine clinically relevant changes and their timing and whether the use of the algorithm improves patient prognosis and/or treatment cost-effectiveness. Still, we believe that a change in risk of death to worse or better informs clinicians about the impact of given pharmaceutical or interventional treatments.

The algorithm performed slightly better in the Swedish cohort than in the eICU cohort. The performance metrics improved clearly over time in the Swedish cohort while they were more stable in the eICU cohort. The reasons for these differences are speculative. For example, the prevalences of high-energy motor vehicle accidents, assaults and gunshot injuries are lower in the Nordic countries than in the USA^18–20^. Though, patients with penetrating brain injury were excluded from the training cohort and the Swedish validation cohort^17^. Hence, it is possible that patients in the eICU cohort suffered from more severe and etiologically different brain injuries than patients in the Finnish training and Swedish validation cohorts, partly explaining the differences in algorithm performance. Although patients were younger in the eICU cohort, they had a more than doubled mortality rate (30 vs. 13%). Moreover, patients in the eICU cohort had lower GCS scores on admission than patients in the Stockholm cohort. Therefore, these differences between the cohorts may contribute to the differences in algorithm performance between the eICU and Stockholm cohorts. In addition, factors such as socioeconomic, ethnic and insurance status differences might have affected the mortality rates in the eICU cohort, while these factors presumably have less importance for patient outcomes in the Nordic countries that use government-funded single-payer healthcare systems^21–23^. Nevertheless, despite considerable demographic differences, we observed that the ICP-MAP-CPP algorithm performed well in both validation cohorts, which suggests that this prediction tool can be expected to perform well in a real-world setting.

There are some limitations that should be highlighted. First, as the eICU database did not contain data regarding 30-day mortality, we relied on in-hospital mortality. However, among hospital survivors in the eICU cohort, the median hospital length of stay was 21 days compared to 5 days among hospital non-survivors. Thus, the likelihood of patients dying between hospital discharge and 30 days is small and its effect on our results is likely non-relevant. Still, whether some of the patients did die between hospital discharge and 30 days they would be misclassified by the algorithm. This could falsely increase the number of false positives in the eICU cohort (i.e., a patient is alive at discharge but dies within 30 days when the algorithm predicted death). Second, we used all-cause 30-day mortality and not specifically neurological cause death. Although it is possible that the direct cause of death is a non-neurological cause, the death will still be indirectly injury related. Moreover, derangements in ICP correlate poorly with non-neurological causes of death and, thus, these may not be captured by the algorithm (i.e., increasing false negatives)^11,24^. Third, prospective validation with treatment data for more than 5 days is necessary to assess the validity of the model over the first 120 h. Based on the Stockholm cohort, predictions might improve with an increasing amount of big data, i.e., with increasing days of monitoring. Fourth, although the included variables (age, MAP, ICP) are widely available in ICUs all over the world, the algorithm is developed and validated in three high-income countries (Finland, Sweden, USA). The burden and mortality of TBI is highest in low- and middle-income countries^1,25^. Thus, it would be of importance to test the algorithm in such settings, where the potential benefits may be more substantial. Such data were unfortunately not available for this study and might be challenging to obtain in large numbers. Noteworthy, the retrained algorithm included somewhat different features than the original ICP-MAP-CPP algorithm^17^. The original ICP-MAP-CPP algorithm included 15 features, of which 6 are included in the retrained algorithm. Further, in the original algorithm, there were 2 CPP-based features, 6 ICP-based features and 6 MAP-based features compared to 4 CPP-based features, 6 ICP-based features and 3 MAP-based features in the retrained algorithm. Still, the performance of the original and retrained model was as good as identical.

High ICP is associated with a higher risk of death^5–7,11,24^. Nevertheless, there is wide variation in the practice of ICP monitoring in Europe^26^, Australia and New Zealand^27^, Canada^28^, and the US^29^. The ICP-MAP-CPP algorithm offers a few properties that could enable its clinical implementation with the potential to improve quality of care and patient outcomes, as well as standardize treatment policies of severely injured TBI patients with a prolonged ICU stay. First, as a dynamic prediction model based on more than 60,000 h of ICP and CPP data to quantify the effect of ICP and CPP on patient outcomes, the algorithm provides repeated simple numbers informing the clinicians about the developing trend of the patient’s clinical status. Therefore, the algorithm is particularly useful in the case of prolonged treatment when decisions to continue or withdraw the treatment become perhaps more and more challenging. In this context, however, it is important that the algorithm does not suggest any specific treatment approach or policy. Second, the ICP-MAP-CPP algorithm is based upon averaged low-frequency data (1 to 5-min medians) from variables that are all widely available in ICUs around the world. The use of low-frequency data overcomes some major challenges in terms of data interruption and artifact signals, all of which are frequent during ICU stay. Artifacts and interruptions may occur, for example, in association with, e.g., probe zeroing, catheter flushing, patient position changes, emergency medical interventions and radiological investigations. Thus, the algorithm is designed to function even in clinical situations with repeatedly disrupted data. Third, the algorithm is completely automatized and does not require any manual input, which minimizes the risk of human errors. Fourth, the outcome of interest is 30-day mortality. Mortality is a hard and crude outcome, which is not subject to interpretability, or cultural or societal differences. Further, 30-day mortality is short enough to be regarded as a direct injury related endpoint. However, predicting long-term TBI-related neurological outcomes using more granular functional outcome metrics could be one of the future aims. Fifth, the algorithm is calibrated (threshold 50%) to minimize the risk of giving false positive predictions (i.e., predicting death when the patient survives) at the cost of false negatives (i.e., predicting survival when the patient dies). This is to minimize the risk of a scenario where a clinician would withdraw critical treatment based on false positive predictions. Similarly, as the reliability of the predictions increases with increasing data, the algorithm use could be formally restricted for prolonged ICU treatments, i.e., patients monitored for at least 3 days or longer. This would further minimize the risk of early treatment withholds or withdrawals based on algorithm predictions.

## Methods

### Study design and patient cohorts

We conducted a multicenter observational retrospective study including adult patients (16 years or older) who were acutely admitted to the intensive care unit (ICU) due to TBI. We only included patients that had ICP data for at least 24 h.

The study was approved by the research committees of Helsinki university Hospital (HUS/182/2021), Kuopio University Hospital (507T013), Turku University Hospital (TP2/008/18), Tampere University Hospital (R18525) and Karolinska University Hospital (Dnr 2020-05227). Access to the eICU database was granted through https://physionet.org/.

### Training cohort

The training cohort consisted of patients with TBI treated in four university hospital ICUs in Finland (Helsinki University Hospital, Helsinki Finland [2010–2019], Kuopio University Hospital, Kuopio Finland [2004–2013], Turku University Hospital, Turku, Finland [2003–2013]) and Tampere University Hospital, Tampere, Finland [2007–2017]). Together, these tertiary ICUs cover approximately 85% of Finland’s population. The TBI treatment protocols in the Finnish ICUs are similar and based upon the most recent Brain Trauma Foundation guideline^10,30,31^.

Clinical data were collected and manually verified from electronic health care records. ICU data were collected from electronic databases (“PICIS Critical Care Suite”, PICIS Clinical Solutions, Barcelona, Spain and “Centricity Critical Care Clinisoft”, GE Healthcare, Chicago, Ill, USA). From the electronic ICU databases, we collected ICP, MAP and CPP in 1 to 5-min median values (as locally stored) and rounded them to the nearest full minute time resolution.

### External validation cohorts

For the external validation, we used a cohort of patients with TBI treated in the neurosurgical intensive care unit at Karolinska university hospital, Stockholm, Sweden (referred to as “Stockholm cohort”) and a cohort of patients with TBI from the eICU database, coming from several ICUs in the USA (referred to as “eICU cohort”)^32,33^.

The patients in the Stockholm cohort were all admitted to the neurosurgical ICU of Karolinska University Hospital in Stockholm, Sweden during 2006–2019. The unit has a catchment area of approximately 2.5 million people and is the only unit providing neurosurgical and neurointensive care in the Stockholm metropolitan area (including the island of Gotland). Clinical and outcome data were collected prospectively, and intensive care data were stored electronically in electronic databases (“Centricity Critical Care Clinisoft”, GE Healthcare, Chicago, Ill, USA). ICP, MAP and CPP values were stored in 1 to 2-minute median values. The treatment protocol at the neurosurgical ICU at Karolinska University Hospital is similar to that of the Finnish ICUs’ cohort^34^.

The patients in the eICU cohort were extracted from the PhysioNet database after obtaining proper permissions [9,10]. The eICU database is a telehealth system developed by Philips Healthcare. The eICU database includes over 200,000 patients from several ICUs in the USA treated during 2014–2015. From the eICU database, we extracted patients with an APACHE IV (Acute Physiology and Chronic Health Evaluation)^35^ diagnosis indicating TBI (see Supplementary Table 4 for diagnosis list) and were monitored for ICP for at least 24 h. In the eICU database, ICP, MAP and CPP values were stored in 5-minute median values.

### Definition of the outcome

The algorithm was developed to predict the risk of 30-day all-cause mortality from admission^17^. We used 30-day mortality as the primary outcome of interest when training the algorithm and for testing the algorithm in the Stockholm cohort. For external validation in the eICU cohort, we used in-hospital mortality, as 30-day mortality was not available. In the Stockholm cohort, we assessed one-year functional outcome for false positives (unfavorable functional outcome defined as a Glasgow Outcome Scale of 1–3)^36^.

### Algorithm description and retraining

The algorithm has previously been developed in a cohort consisting of 472 patients with TBI from three Finnish tertiary ICUs^17^. The original code is open-sourced and be found at https://static-content.springer.com/esm/art%3A10.1038%2Fs41598-019-53889-6/MediaObjects/41598_2019_53889_MOESM1_ESM.pdf. The code for the retraining and testing can be found at https://github.com/ralleraj/aip_tbi. The ICP-MAP-CPP algorithm was developed as a fully automated and objective dynamic algorithm to predict 30-day mortality based upon ICP, MAP and CPP. The algorithm uses a logistic regression approach with rolling time windows. Before retraining, extreme measurements (ICP > 100 mmHg or < 0 mmHg, MAP > 150 mmHg or < 20 mmHg) were excluded. Features were designed as means from the first 24 h time-window (begin), means from the last 8 h time-window (end), linear trend coefficients from the last time-window (coef), minimum values from the last time-window (min), maximum values from the last time-window (max), means of differences from the last time-window (diff), variances from the last time-window (var) and mean values from the last time-window (avg). Specific ICP features were designed to capture the percentage of data points being higher than 20 mmHg (ht20) and lower than 10 mmHg (lt10). Specific MAP features were designed to capture the percentage of data points being higher than 120 mmHg (ht120), as a measure of severe arterial hypertension. Features were designed to capture the trends of the most extreme values in terms of the highest 90th percentile (q90) and the lowest 10th percentile (q10). Finally, 54 features (+age in deciles, “agec”) were considered for the ICP-CPP-MAP model (Supplementary Table 2). The features’ regression coefficients (Supplementary Table 3) are constant, and the features’ values were calculated in 4 h rolling time-windows. The optimal features and number of features were chosen using a stratified cross-validation technique. Thus, the included features might differ depending on how the folds are randomized. The algorithm gives a prediction ranging from 0% to 100% every 8 h following the first 24 h.

Here, we retrained the algorithm with additional available data in an extended training cohort including 686 patients from four Finnish tertiary ICUs. A five-fold cross-validation technique was used for the internal validation.

### Statistical analysis

We conducted all analyses using Stata version 15 (StataCorp, College Station, TX) and Google Colaboratory (Mountain View, CA, USA). In Google Colaboratory, we used python 3.7.12 for the retraining and validation of the algorithm. The following libraries were used scikit-learn (version 1.0.1), tqdm (version 4.62.3), pandas (version 1.1.5), numpy (version 1.19.5), matplotlib (version 3.2.2), seaborn version 0.11.2), joblib (version 1.1.0) and bayesian-optimization (version 1.2.0).

Continuous data were tested for skewness using the Kolmogorov–Smirnov test. Normally distributed data were presented as means with standard deviations and nonparametric data were presented as medians with interquartile ranges. Differences in categorical variables between groups were tested using a two-sided chi-square test and differences between non-parametric continuous variables were tested using a Wilcoxon rank-sum test.

We externally validated the trained algorithm in the Stockholm and eICU cohorts. To assess algorithm performance, we calculated the area under the receiver operating characteristic curve (AUC), the area under the precision-recall curve (AUPRC) and accuracy as functions of time. The AUC is a combination of sensitivity and specificity and tests the discriminatory power of the algorithm, i.e., what is the likelihood that a patient with the outcome (death) has a higher risk of death than a patient without the outcome (alive).

We calculated the rate of false positives (i.e., patients that were predicted to have a fatal outcome but survived, fp) and false negatives (patients that were predicted to survive but had a fatal outcome, fn) at every prediction time-point. Ideally, the fp and fn rates are low, but for an algorithm that deals with real-time prognoses of death, avoidance of fp is crucial to minimize the risk of withdrawing active treatment (Fig. 1).

AUPRC is a combination of precision and recall. Precision depicts how well the classifier manages not to label a negative sample as positive. Precision is defined: tp/(tp + fp), where tp is the number of true positives and fp the number of false positives. Recall is defined as: tp/(tp + fn), where fn is the number of false negatives. Hence, it represents the ability of the classifier to find all the positive samples. Accuracy is the fraction of correctly classified samples; the maximum value for it is 1 if normalization is used, which is the case in this study.

If the predicted risk of death was higher than 50% and the patient survived, he/she was considered an fp. If the predicted risk of death was lower than 50% and the patient died, he/she was considered an fn [5]. The fp and fn rates were plotted as a function of time. We also increased and decreased the 50% threshold to 75% and 25%, respectively, to demonstrate its effect on the fp and fn rates.

### Reporting summary

Further information on research design is available in the Nature Research Reporting Summary linked to this article.

## Supplementary information

Reporting Summary

Supplementary Information

## Data Availability

Finnish healthcare data for secondary use can be obtained through FINDATA (Social and Health Data Permit Authority according to the Secondary Data Act. Access to the eICU database can be obtained through https://physionet.org/. Swedish healthcare data cannot be shared openly. Data can be made available upon request on a case-by-case basis as allowed by the legislation and ethical permits. Requests for access can be made to the Karolinska Institutet’s Research Data Office at rdo@ki.se.

## Code availability

The code for the ICP-MAP-CPP algorithm is available at https://github.com/ralleraj/aip_tbi and https://static-content.springer.com/esm/art%3A10.1038%2Fs41598-019-53889-6/MediaObjects/41598_2019_53889_MOESM1_ESM.pdf. Included features included and their regression coefficients are shown in Supplementary Table 3.
