# Supplementary material for: Dynamic prediction of mortality after traumatic brain injury using a machine learning algorithm
Source: NPJ Digit Med. 2022 Jul 18;5:96. doi: 10.1038/s41746-022-00652-3 (PMC9293936; doi:10.1038/s41746-022-00652-3)
Supplement: Supplementary file 1 — Reporting Summary [file 41746_2022_652_MOESM1_ESM.pdf]

nature portfolio

Corresponding author(s): Rahul Raj

Last updated by author(s): Jun 30, 2022

Reporting Summary

Nature Portfolio wishes to improve the reproducibility of the work that we publish. This form provides structure for consistency and transparency in reporting. For further information on Nature Portfolio policies, see our Editorial Policies and the Editorial Policy Checklist.

Statistics

For all statistical analyses, confirm that the following items are present in the figure legend, table legend, main text, or Methods section.

n/a	Confirmed	
☐	☒	The exact sample size (*n*) for each experimental group/condition, given as a discrete number and unit of measurement
☐	☒	A statement on whether measurements were taken from distinct samples or whether the same sample was measured repeatedly
☐	☒	The statistical test(s) used AND whether they are one- or two-sided<br>*Only common tests should be described solely by name; describe more complex techniques in the Methods section.*
☐	☒	A description of all covariates tested
☐	☒	A description of any assumptions or corrections, such as tests of normality and adjustment for multiple comparisons
☐	☒	A full description of the statistical parameters including central tendency (e.g. means) or other basic estimates (e.g. regression coefficient) AND variation (e.g. standard deviation) or associated estimates of uncertainty (e.g. confidence intervals)
☐	☒	For null hypothesis testing, the test statistic (e.g. *F*, *t*, *r*) with confidence intervals, effect sizes, degrees of freedom and *P* value noted<br>*Give P values as exact values whenever suitable.*
☒	☐	For Bayesian analysis, information on the choice of priors and Markov chain Monte Carlo settings
☐	☒	For hierarchical and complex designs, identification of the appropriate level for tests and full reporting of outcomes
☒	☐	Estimates of effect sizes (e.g. Cohen's *d*, Pearson's *r*), indicating how they were calculated

Our web collection on statistics for biologists contains articles on many of the points above.

Software and code

Policy information about availability of computer code

Data collection	Physionet eICU; Centricity Critical Care Clinisoft", GE Healthcare, Chicago, Ill, USA; PICIS Critical Care Suite, PICIS Clinical Solutions, Barcelona, Spain; Centricity Critical Care Clinisoft, GE Healthcare, Chicago, Ill, USA
Data analysis	Stata version 15 (StataCorp, College Station, TX) and Google Colaboratory (Mountain View, CA, USA). In Google Colaboratory: python 3.7.12. The following libraries were used scikit-learn (version 1.0.1), tqdm (version 4.62.3), pandas (version 1.1.5), numpy (version 1.19.5), matplotlib (version 3.2.2), seaborn version 0.11.2), joblib (version 1.1.0) and bayesian-optimization (version 1.2.0).

For manuscripts utilizing custom algorithms or software that are central to the research but not yet described in published literature, software must be made available to editors and reviewers. We strongly encourage code deposition in a community repository (e.g. GitHub). See the Nature Portfolio guidelines for submitting code & software for further information.

Data

Policy information about availability of data

All manuscripts must include a data availability statement. This statement should provide the following information, where applicable:

- Accession codes, unique identifiers, or web links for publicly available datasets
- A description of any restrictions on data availability
- For clinical datasets or third party data, please ensure that the statement adheres to our policy

Finnish healthcare data for secondary use can be obtained through FINDATA (Social and Health Data Permit Authority according to the Secondary Data Act. Access

to the eICU database can be obtained through https://physionet.org/. Swedish healthcare data cannot be shared openly. Data can be made available upon request on a case-by-case basis as allowed by the legislation and ethical permits. Requests for access can be made to the Karolinska Institutet's Research Data Office at rdo@ki.se.

Human research participants

Policy information about studies involving human research participants and Sex and Gender in Research.

Reporting on sex and gender	N/A
Population characteristics	Patients with traumatic brain injury being monitored for intracranial pressure and treated in the intensive care unit
Recruitment	Retrospective register-based study
Ethics oversight	The study was approved by the research committees of Helsinki university Hospital (HUS/182/2021), Kuopio University Hospital (507T013), Turku University Hospital (TP2/008/18), Tampere University Hospital (R18525) and Karolinska University Hospital (Dnr 2020-05227). Access to the eICU database was granted through https://physionet.org/.

Note that full information on the approval of the study protocol must also be provided in the manuscript.

Field-specific reporting

Please select the one below that is the best fit for your research. If you are not sure, read the appropriate sections before making your selection.

☒ Life sciences ☐ Behavioural & social sciences ☐ Ecological, evolutionary & environmental sciences

For a reference copy of the document with all sections, see nature.com/documents/nr-reporting-summary-flat.pdf

Life sciences study design

All studies must disclose on these points even when the disclosure is negative.

Sample size	No predefined sample size was calculated
Data exclusions	Age under 18y, ICP monitoring for less than 24h, extreme data points: ICP >100 mmHg or <0 mmHg, MAP >150 mmHg or <20 mmHg
Replication	All results were carried out in two external validation cohorts sepratately
Randomization	N/A
Blinding	N/A

Reporting for specific materials, systems and methods

We require information from authors about some types of materials, experimental systems and methods used in many studies. Here, indicate whether each material, system or method listed is relevant to your study. If you are not sure if a list item applies to your research, read the appropriate section before selecting a response.

Materials & experimental systems

n/a	Involved in the study	
☒	☐	Antibodies
☒	☐	Eukaryotic cell lines
☒	☐	Palaeontology and archaeology
☒	☐	Animals and other organisms
☐	☒	Clinical data
☒	☐	Dual use research of concern

Methods

n/a	Involved in the study	
☒	☐	ChIP-seq
☒	☐	Flow cytometry
☒	☐	MRI-based neuroimaging

Clinical data

Policy information about clinical studies

All manuscripts should comply with the ICMJE guidelines for publication of clinical research and a completed CONSORT checklist must be included with all submissions.

Clinical trial registration	N/A
Study protocol	N/A. External validation study. Original study: Sci Rep. 2019 Nov 27;9(1):17672. doi: 10.1038/s41598-019-53889-6.
Data collection	Helsinki University Hospital, Helsinki Finland [2010–2019], Kuopio University Hospital, Kuopio Finland [2004–2013], Turku University Hospital, Turku, Finland [2003–2013]) and Tampere University Hospital, Tampere, Finland [2007-2017], Karolinska University Hospital [2006–2019], eICU [2014–2015)
Outcomes	All cause 30-day mortality
