# Supplementary material for: Dynamic prediction of mortality after traumatic brain injury using a machine learning algorithm
Source: NPJ Digit Med. 2022 Jul 18;5:96. doi: 10.1038/s41746-022-00652-3 (PMC9293936; doi:10.1038/s41746-022-00652-3)

Supplementary Information

Table of Contents

Supplementary Table 1: Patient characteristics in the training cohort according to hospital 2

Supplementary Table 2: All considered features 4

Supplementary Table 3: Included features and regression coefficients 5

Supplementary Figure 1: Difference in the mean level of intracranial pressure between 30-day survivors and non-survivors in the cohorts 7

Supplementary Figure 2: Recursive feature elimination. 8

Supplementary Figure 3: Feature correlation matrix 9

Supplementary Figure 4: The rate of false positives and false negatives in the training cohort. 10

Supplementary Figure 5: Threshold testing in the Stockholm cohort. 11

Supplementary Figure 6: Threshold testing in the eICU cohort. 12

Supplementary Table 1: Patient characteristics in the training cohort according to hospital

Variable	Helsinki UH (n=248)	Kuopio UH (n=123)	Tampere UH (n=155)	Turku UH (n=160)
**Age**, median (IQR)	44 (27, 57)	55 (41, 63)	39 (23, 59)	44 (27, 61)
**Female sex**	55 (22%)	21 (17%)	31 (20%)	28 (18%)
**Admission GCS score**				
3 to 8	187 (75%)	87 (71%)	96 (62%)	95 (59%)
9 to 12	53 (22%)	21 (17%)	38 (25%)	45 (28%)
13 to 15	8 (3%)	15 (12%)	21 (13%)	20 (13%)
**Admission motor score**				
None	91 (37%)	28 (23%)	38 (25%)	29 (18%)
Extension	8 (3%)	9 (7%)	9 (6%)	8 (5%)
Abnormal flexion	10 (4%)	7 (6%)	4 (2%)	14 (9%)
Normal flexion	35 (14%)	31 (25%)	31 (20%)	27 (17%)
Localizes/obeys	104 (42%)	48 (39%)	73 (47%)	82 (51%)
**Pupillary light reactivity**				
None react	19 (8%)	5 (4%)	2 (1%)	6 (4%)
One reacts	46 (18%)	18 (15%)	17 (11%)	19 (12%)
Both react	183 (74%)	100 (81%)	136 (88%)	135 (84%)
**Hypoxia**	52 (21%)	23 (19%)	15 (10%)	20 (13%)
**Hypotension**	39 (16%)	12 (10%)	9 (6%)	14 (9%)
**Marshall CT class**				
DI I	0 (0%)	4 (3%)	3 (2%)	5 (3%)
DI II	67 (27%)	23 (19%)	104 (66%)	66 (41%)
DI III	51 (21%)	22 (18%)	23 (15%)	25 (16%)
DI IV	11 (4%)	4 (3%)	4 (3%)	6 (4%)
EML/NEML	111 (48%)	70 (57%)	21 (14%)	58 (36%)
**Traumatic SAH**	204 (82%)	65 (53%)	107 (69%)	115 (72%)
**Epidural hematoma**	24 (10%)	11 (9%)	5 (3%)	17 (11%)
**Craniotomy and hematoma evacuation**	109 (44%)	77 (63%)	31 (20%)	56 (35%)

**Decompressive craniectomy**	38 (15%)	19 (15%)	34 (22%)	22 (14%)
Primary	20 (8%)	8 (6%)	16 (10%)	4 (3%)
Secondary	18 (7%)	11 (9%)	18 (12%)	18 (11%)
**External ventricular drain**	37 (15%)	55 (45%)	11 (7%)	11 (7%)
**ICU length of stay** (days), median (IQR)	10 (5, 14)	4 (3-8)	10 (6, 16)	9 (5, 16)
**30-day mortality**	37 (15%)	26 (21%)	18 (12%)	39 (24%)

Hypoxia defined as any prehospital spO2 value <90%
Hypotension defined as any prehospital systolic blood pressure value <90 mmHg

Abbreviations: CT=Computer Tomography, DI=Diffuse Injury, EML=Evacuated Mass Lesion (>25 cm^3), NEML=Non-Evacuated Mass Lesion (>25 cm^3), GCS=Glasgow Coma Scale, IQR=Interquartile Range, UH=University Hospital

Supplementary Table 2: All considered features

1. agec
2. icp_begin
3. icp_end
4. icp_coef
5. icp_q90_begin
6. icp_q90_end
7. icp_q90_coef
8. cpp_var_begin
9. cpp_var_end
10. cpp_var_coef
11. map_begin
12. map_end
13. map_coef
14. map_q90_begin
15. map_q90_end
16. map_q90_coef
17. map_q10_begin
18. map_q10_end
19. map_q10_coef
20. map_diff_begin
21. map_diff_end
22. map_diff_coef
23. cpp_diff_begin
24. cpp_diff_end
25. cpp_diff_coef
26. icp_ht20_begin
27. icp_ht20_end
28. icp_ht20_coef
29. icp_var_begin
30. icp_var_end
31. icp_var_coef
32. map_ht120_begin
33. map_ht120_end
34. map_ht120_coef
35. map_var_begin
36. map_var_end
37. map_var_coef
38. cpp_begin
39. cpp_end
40. cpp_coef
41. icp_diff_begin
42. icp_diff_end
43. icp_diff_coef
44. icp_lt10_begin
45. icp_lt10_end
46. icp_lt10_coef
47. cpp_q10_begin
48. cpp_q10_end
49. cpp_q10_coef
50. icp_q10_begin
51. icp_q10_end
52. icp_q10_coef
53. cpp_q90_begin
54. cpp_q90_end
55. cpp_q90_coef

Abbreviations:
- agec = age category (20, 20-29, 30-39, 40-49, 50-59, 60-69, 70-79, 80-89, 90-99 years)
- icp = intracranial pressure
- cpp = cerebral perfusion pressure
- map = mean arterial pressure
- mr = motor response
- er = eye response
- begin = mean value from the first derived 24-hour time-window
- end = mean value from the last derived 8 hours
- coef = slope of the linear coefficient from the start of the derived time-window up to the time of the
- prediction
- min = minimum value in the derived time-window
- max = maximum value in the derived time-window
- diff = mean of differences between consequent values in the derived time-window
- var = variance in the derived time-window
- avg = mean value in the derived time-window
- q90 = 90th percentile in the derived time-window
- q10 = 10th percentile in the derived time-window
- ht20 = percentage of measurement points being higher than 20 mmHg in the derived time-window
- ht120 = percentage of measurement points being higher than 120 mmHg in the derived time-window
- lt10 = percentage of measurement points being lower than 10 mmHg in the derived time-window

Supplementary Table 3: Included features and regression coefficients

**Feature abbreviation**	**Coefficient (SD)**
agec	0.96 (0.14)
cpp_diff_begin	-0.95 (0.41)
cpp_diff_coef	1.42 (0.83)
cpp_end	0.38 (0.15)
cpp_var_coef	-1.12 (0.62)
icp_coef	0.83 (0.66)
icp_diff_coef	-0.77 (0.41)
icp_diff_end	-0.41 (0.33)
icp_end	1.13 (0.22)
icp_q90_coef	-1.00 (0.66)
icp_var_coef	0.57 (0.36)
map_diff_begin	0.60 (0.40)
map_diff_coef	-1.78 (0.81)
map_var_coef	1.65 (0.61)

A positive coefficient value indicates that a higher feature value increases the risk of 30-day mortality and a negative coefficient value indicates that a higher feature value decreases the risk of 30-day mortality.

Abbreviations:
agec=age categories in deciles
cpp=cerebral perfusion pressure,
map=mean arterial pressure,
icp=intracranial pressure,
end = mean value from the last derived 8 hours,
diff=mean of differences between consequent values in the derived time-window,
begin=mean value from the first derived 24-hour time-window,
end=mean value from the last derived 8 hours,
coef=slope of the linear coefficient from the start of the derived time-window up to the time of the prediction,
var=variance in the derived time-window,
q90=90th percentile in the derived time-window

Supplementary Table 4: Included Acute Physiology and Chronic Health Evaluation (APACHE) diagnoses from the eICU database

Hematoma subdural, surgery for
Head only trauma
Hematoma-epidural, surgery for
Head/chest trauma
Head/multiple trauma
Head/spinal trauma
Head/extremity trauma
Head/face trauma
Head/abdomen trauma
Head/pelvis trauma
Abbreviations: APACHE, Acute Physiology and Chronic Health Evaluation

Supplementary Figure 1: Difference in the mean level of intracranial pressure between 30-day survivors and non-survivors in the cohorts

At the top the training cohort, in the middle the Stockholm cohort and at the bottom the eICU cohort. Means calculated in 12 h windows. Shown with 1 standard deviation.

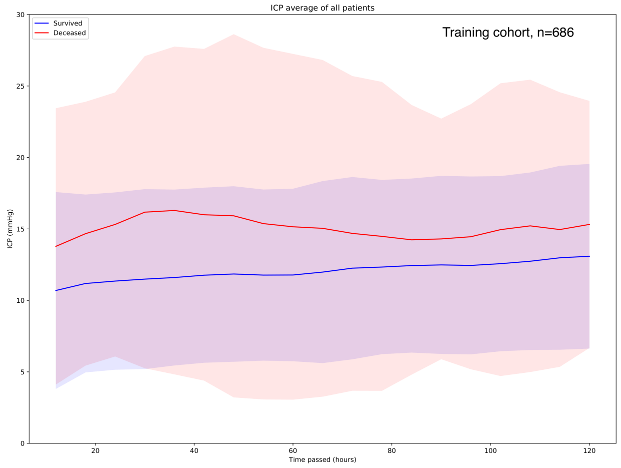

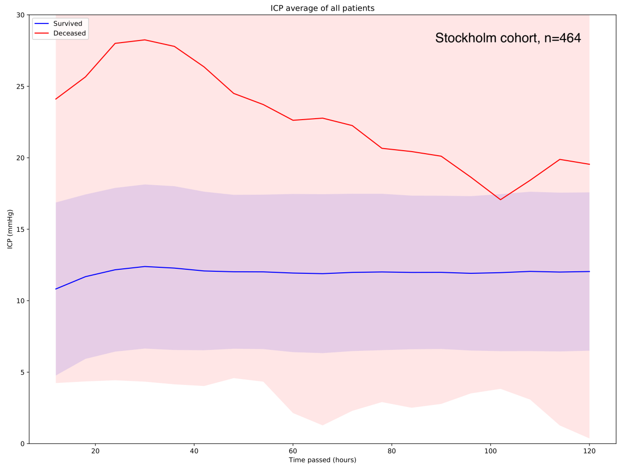

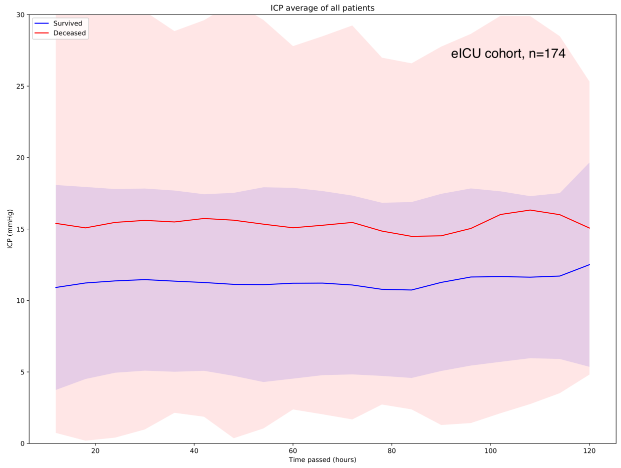

Supplementary Figure 2: Recursive feature elimination.

When retraining the algorithm, the recursive feature elimination gave an optimal of 14 features to be included.

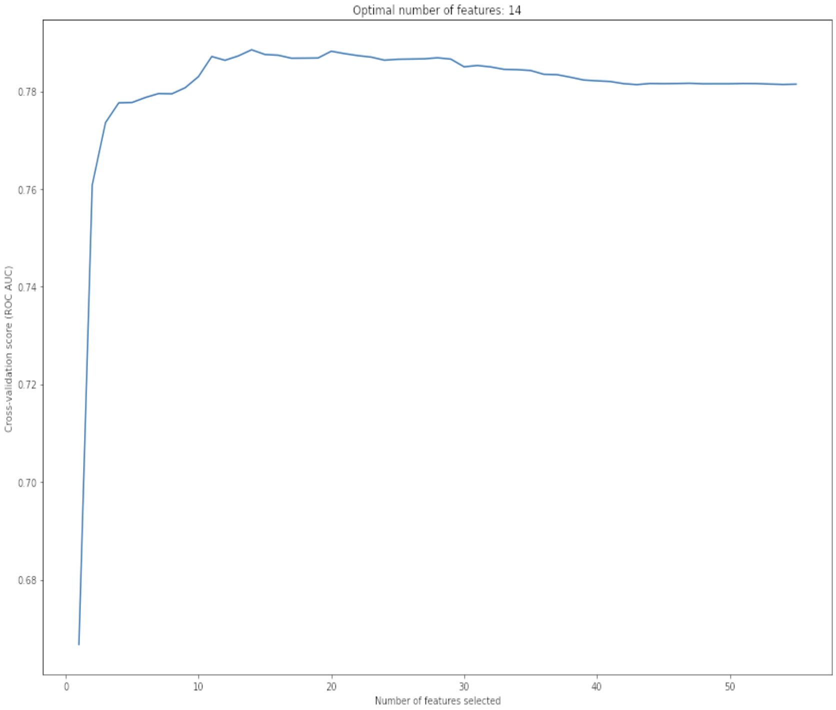

Supplementary Figure 3: Feature correlation matrix

Correlation matrix between included features when retraining the algorithm. Y-axis features in descending order: agec, icp_end, icp_coef, cpp_end, icp_var_coef, map_var_coef, cpp_var_coef, icp_diff_end, icp_diff_coef, map_diff_begin, map_diff_coef, cpp_diff_begin, cpp_diff_coef, icp_q90_coef

X-axis features from left to right: agec, icp_end, icp_coef, cpp_end, icp_var_coef, map_var_coef, cpp_var_coef, icp_diff_end, icp_diff_coef, map_diff_begin, map_diff_coef, cpp_diff_begin, cpp_diff_coef, icp_q90_coef

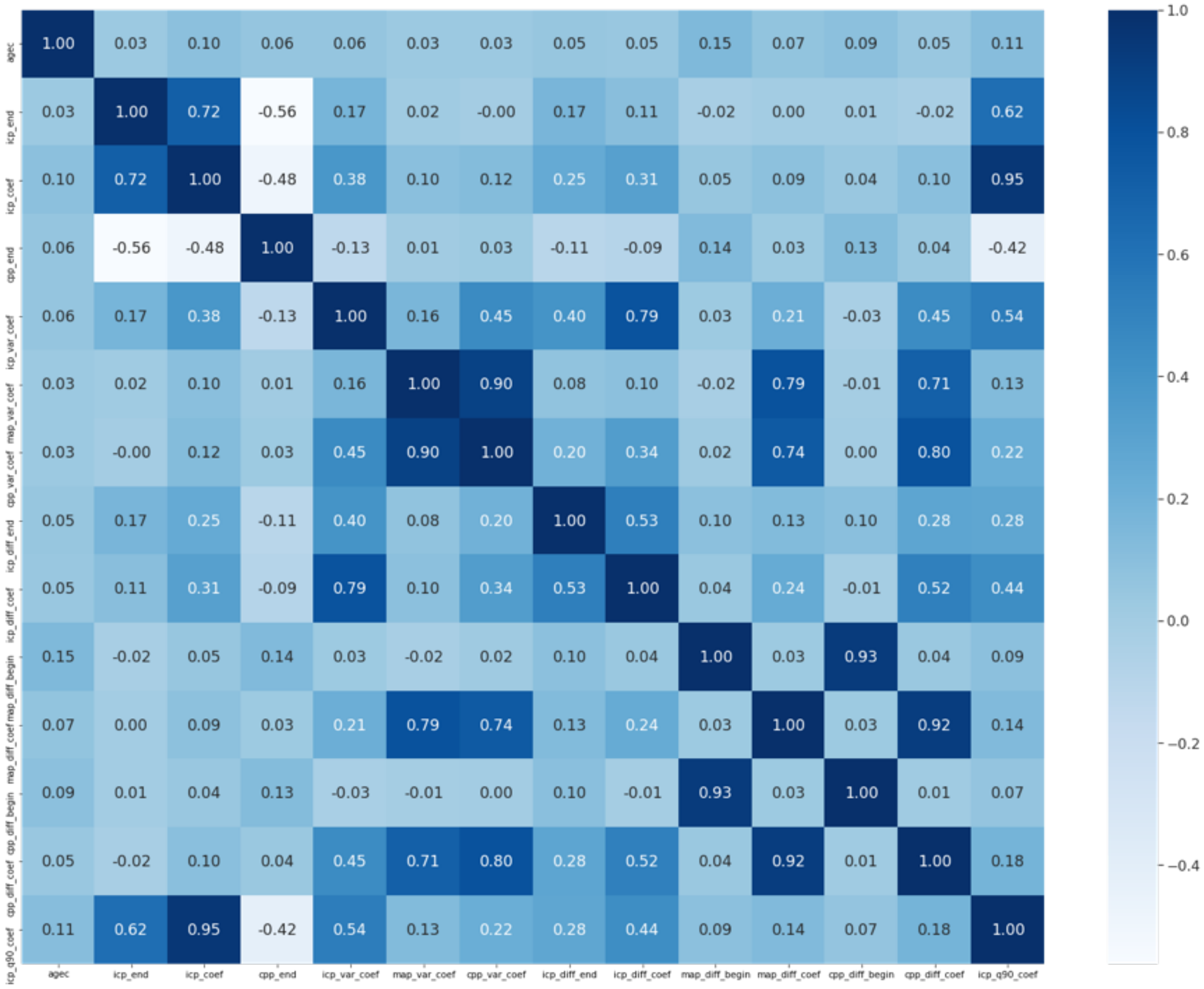

Supplementary Figure 4: The rate of false positives and false negatives in the training cohort.

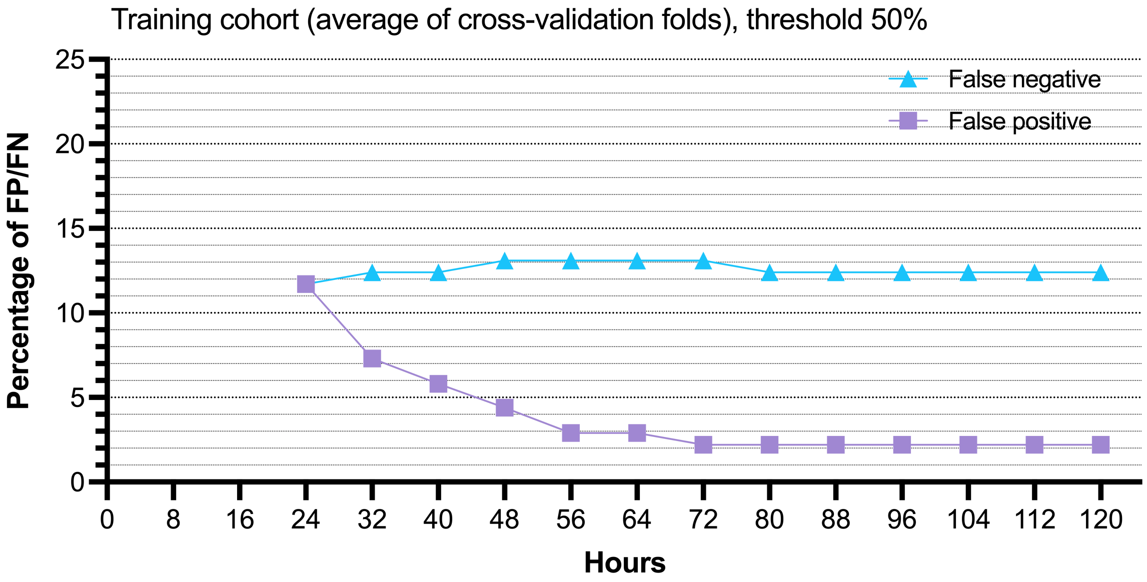

Supplementary Figure 5: Threshold testing in the Stockholm cohort.

Decreasing the prediction threshold from 50% to 25% increased the number of false positives but decreased the number of false negatives. Conversely, increasing the prediction threshold from 50% to 75% decreased the number of false positives but increased the number of false negatives.

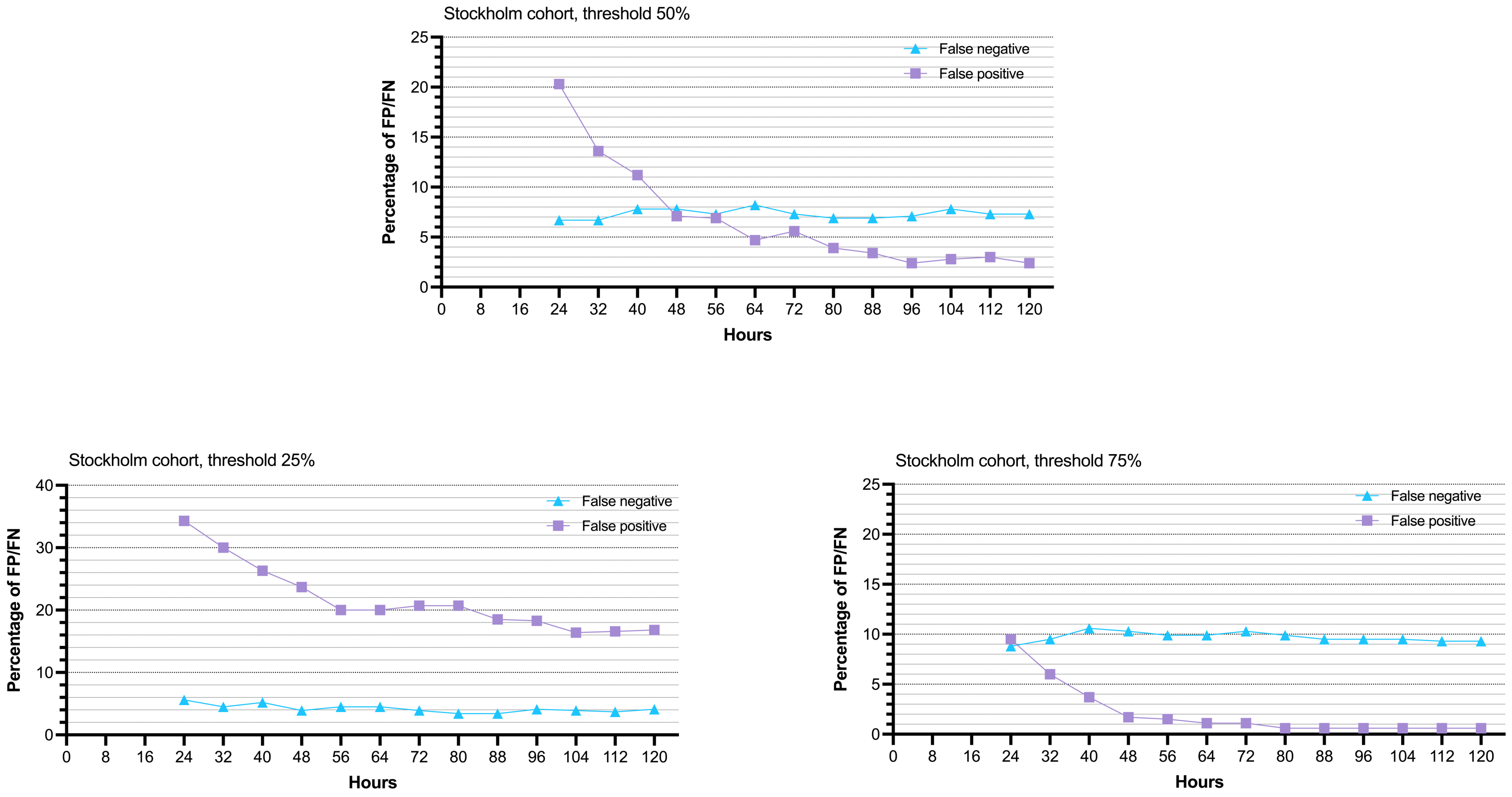

Supplementary Figure 6: Threshold testing in the eICU cohort.

Decreasing the prediction threshold from 50% to 25% increased the number of false positives but decreased the number of false negatives. Conversely, increasing the prediction threshold from 50% to 75% decreased the number of false positives but increased the number of false negatives.

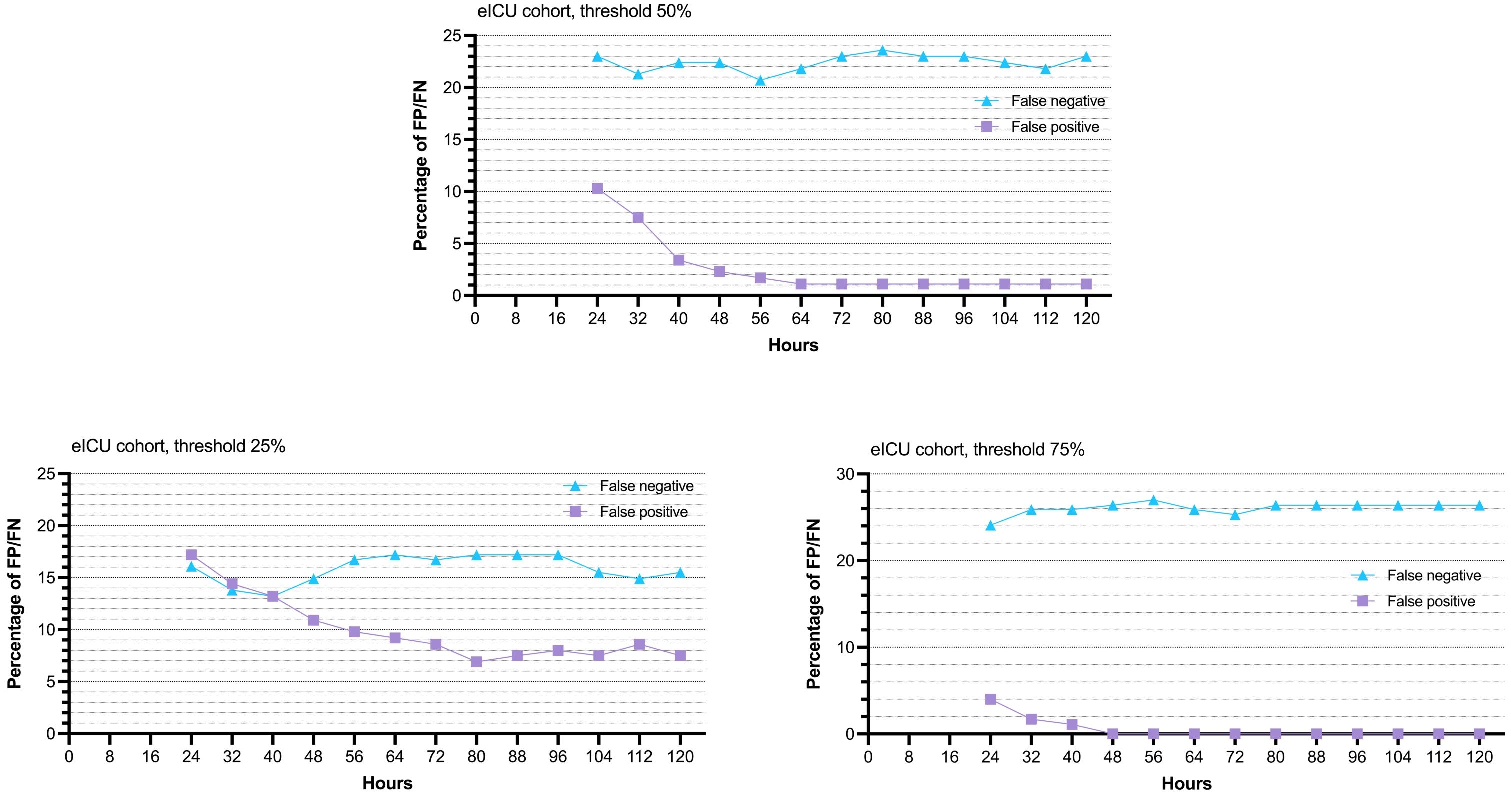

Supplement: Supplementary file 2 — Supplementary Information [file 41746_2022_652_MOESM2_ESM.pdf]
